# Evaluation of the Efficiency of Basic Sanitation Integrated Management in Brazilian Municipalities

**DOI:** 10.3390/ijerph17249244

**Published:** 2020-12-10

**Authors:** Alvaro Cavalcanti, Arthur Teixeira, Karen Pontes

**Affiliations:** 1 Federal Institute of Education, Science and Technology of Paraíba, 58015-020 Paraíba, Brazil; 2 Graduate Program of Industrial Engineering, Federal University of Bahia, 40210-630 Bahia, Brazil; arthurtc@ufba.br (A.T.); karenpontes@ufba.br (K.P.); 3 Department of Mechanical Engineering, Federal University of Bahia, 40210-630 Bahia, Brazil; 4 Department of Chemical Engineering, Federal University of Bahia, 40210-630 Bahia, Brazil

**Keywords:** technical efficiency, basic sanitation sector, Brazilian municipalities, data envelopment analysis

## Abstract

This study aims to evaluate the level of technical efficiency of companies that perform the integrated management of basic sanitation in Brazilian municipalities. A Multiple Data Envelopment Analysis (M-DEA) model was applied to estimate the performance of water supply and sewage services in 1628 municipalities covering more than 56% of the Brazilian population, identifying the factors that most influence the efficiency of the sector in the years 2008 and 2016. The M-DEA methodology is an extension of Data Envelopment Analysis (DEA) with multiple DEA executions considering all combinations of inputs and outputs to calculate efficiency scores. The methodology reduces possible biases in the selection of resources and products of the model, ability to support decision-making in favor of improvements in the sector′s efficiency based on national regulatory framework. The analyses show that the companies analyzed can increase their operating results and attendance coverage by more than 60%, given the current levels of infrastructure, human and financial resources in the sector. Based on the simulation of potential efficiency gains in Brazilian basic sanitation companies, the estimates show that the coverage of the population with access to sanitary sewage would go from the current 59.9% to 76.5%. The evidence found provides indications to subsidize sanitation management in the country at the micro-analytical level, enabling a better competitive position in the sector for the integrated management of basic sanitation and its universalization in Brazil.

## 1. Introduction

Access to treated water and adequate sanitary facilities are the basis of all sanitation infrastructure compatible with environmental sustainability and human rights [1,2,3]. Currently, 2.17 billion people worldwide face a scarcity of treated water and three out of five people live in dwellings without a sewage system, which is equivalent to 29.4% and 60.6% of the world population, respectively [4]. However, the developed countries that are part of the Organization for Economic Cooperation and Development (OECD) show a trend close to universalization of sanitation coverage, in which 4.2% of the population has no access to treated water and only 11.5% has no sewage system [5]. In light of this, international guidelines of the United Nations [2], the World Health Organization [4] and the World Bank [6] highlight the strategic importance of the basic sanitation sector for human health and sustainable development as part of a recent global effort to universalize coverage by 2030.

In Brazil, out of a total of 206.1 million inhabitants [7], 10% of the population do not have access to treated water and 37% of the population live in areas without a sewage network [8], which are lower than the global indicators, although higher than that observed in developed countries. In addition, Brazil has regional distortions that make the national reality much more challenging to fully attend the population. In the northeast and north regions, for example, 17% and 29% of the population, respectively, do not have access to piped water [8]. In the south (4%), southeast (8%) and midwest (7%) regions, these numbers are lower. With regard to sewage collection in the northeast and north regions, 60% and 84% of the population, respectively, live in locations without a sewage collection network; while in the south (39%), southeast (20%), and midwest (37%) regions the percentages are lower [8]. It is known that these regional distortions are due to socioeconomic imbalances between the regions and, consequently, to the greatest deficiencies of basic infrastructure in the poorest regions of Brazil, with emphasis on the low levels of access to piped water and sanitation [9].

In fact, the improvement of water supply and sewage services provides a cost-benefit ratio, since each US$1.00 invested in basic sanitation reduces US$4.30 with expenses on health treatments resulting from the unavailability of these services [10,11]. For Brazil, sanitation benefits are estimated at US$8.93 for every US$1.00 invested in sanitation coverage universalization [10]. Lack of access to drinking water and sanitation are among the top five risk factors for infant mortality worldwide for children under five years of age [12,13]. In addition, the 10% increase in access to domestic sewage is associated with a reduction of 5.7 deaths per 1000 live births [14]. Diseases related to inadequate basic sanitation cause hospitalization of 1 in 10 children hospitalized in that age group in Brazil [15].

There is evidence of the low efficiency of basic sanitation services in Brazil, compared to the results achieved by other countries [16]. The level of annual investment in infrastructure in the Brazilian basic sanitation sector is higher than the world average investment of 0.120% of the gross domestic product (GDP) [17]. In fact, in 2016, the total investment in improving operations and expansion by companies in the sector was responsible for 0.183% of Brazil′s GDP [7,8], of which R$ 1.42 billion by private management (0.029% of GDP) and R$ 7.57 billion (0.154% of GDP) by the public administration [8].

The difficulty in providing public sanitation services, in terms of access and quality, are governance challenges to be overcome in the next two decades, as highlighted by several authors [9,16,18,19,20,21,22,23,24,25]. Studies in the field reveal a growing preoccupation in better understanding the causes and consequences of efficiency problems in the use of infrastructure, human, and financial resources in this sector, in order to boost development and competitiveness between agencies and sanitation companies. The most recent studies admit that the low productivity of Brazilian sanitation companies hinders a better level of coverage and quality of services provided to the population [16,18,21]. Therefore, the identification of public and private companies operating ineffectively proves to be fundamental to guide sector planning in the short and medium terms and strategic actions aimed at their universalization. Here, the term “universalization” is associated with the notion of universality, access to water and sewage services that are provided to the entire Brazilian population, in relation to the fulfillment of Goal 6 of the SDGs [2] and goals A1 and E1 of PLANSAB [26].

The conventional DEA method has been widely used nationally and internationally to measure the efficiency of the basic sanitation sector, however, there is no evidence of a more robust approach (such as M-DEA) applied to the sector, and many of the previous studies deal with water supply and sewage services in isolation [27]. Abbott and Cohen [28] presented a literature review on the measurement of efficiency in public and private companies, already citing the seminal study by Byrnes et al. [29] that uses DEA to analyze the efficiency of water supply in the United States. Subsequent studies with conventional DEA began to reflect the importance of the efficiency of basic sanitation not only to improve the efficiency of the sector itself, but also to subsidize the public policies and decision-making in the public health sector [9,10,11,12,13,14,15,16,17,18,30].

Studies concerning Brazil on the subject are also found in the literature, where a concentration of the use of the conventional DEA approach is identified from a methodological point of view. The results of this approach, however, are sensitive to the choice of resources and products [18,22,24,25,31,32], previous studies have limitations of approach in estimating the efficiency of basic sanitation. Firstly, national and international studies are limited to samples of BSUs by arbitrary criteria of quantity of observations and types of BSU (public, private, regional, local, integrated or not, etc.), like Macedo [18], which adopted only 18 BSUs representative of federation units for the evaluation of productivity in Brazil, and Abbott et al. [33] who used aggregated BSU data from six Australian capitals to assess productivity in the Basic sanitation sector. Second, for the Brazilian results, the existing studies are still few about the integrated management of Brazilian Basic sanitation [22]. In addition, there is no uniformity of inputs (resources) and outputs (products) in the literature, which makes it difficult to compare the sector performance in terms of the robustness of the model and the decision-making power in allocating scarce resources and creating priorities in sector’s planning [20,34]. It is these gaps that corroborate the importance of this research.

The research developed here adopted the M-DEA to classify the public and private companies in the integrated management of the sectors of supply of treated water and sewage, hereinafter referred to in this work as Basic sanitation Units (BSU). This study allows subsidizing the decision making of the managers of BSU based on the measurement of the efficiency, in order to reduce potential waste and maximize the coverage of attendance and the operational results. The chosen methodology is an extension of the conventional DEA computing all possible combinations between the resources and products used in the efficiency calculation, which reduces the discretion of choosing the model variables and the sensitivity of the efficiency scores. It is a modeling contribution to the efficiency assessment literature that applies conventional DEA to basic sanitation results.

The present study considers the results of the water and sewage integrated management in each municipality of the national territory as results of Brazilian sanitation companies, generating a wider set of BSU observations than the literature usually considers when estimating the sector efficiency. In addition, a framework for measuring performance in the basic sanitation sector, for the first time is presented in the literature using the M-DEA model proposed by Stosic and Fittipaldi [35]. This proposal brings direct evidence on the productivity of the Brazilian basic sanitation sector with benchmark BSU indicators to facilitate the implementation at the operational levels and potential gains with the efficiency improving of Brazilian companies, given the current levels of operational and financial resources available. This efficiency estimation model reduces the bias in the selection of resource and product variables and calculates with better context the performance of the BSU in the Brazilian sector, with the possibility of projecting the level of coverage of water and sewage services, having as goal their universalization.

Therefore, there are three main motivations for this work. First, the low performance of the universalization process for these services in Brazil and the persistent inequalities in poorer Brazilian regions. It is worth mentioning here the importance recognized in the literature on basic sanitation for the well-being of the population. Second, the recent global effort to universalize the supply service of treated water by 2030, and sanitary conditions essential for human life and sustainable development goals (SDGs). Third, the need of empirical evidence to guide sector planning in the short and medium terms, with a view to impacting the lives of the population and meeting the basic needs of citizenship and justice, which require commitment at all levels, local, national and global. This study provides an integrated analysis of the sector′s performance from the perspective of productivity improvement ignored until now in Brazilian regulatory frameworks. Thus, the evaluation of the universalization process and the fact that companies are using the resources that will jointly lead to full coverage of water and sewage services becomes a relevant empirical field to improve the level of coverage of services provided to the population and support the SDG vision of universalizing these services in Brazil.

The general objective of this article is to evaluate the level of technical efficiency of the Brazilian basic sanitation companies, considering the relevance of indicators commonly adopted by the literature to measure efficiency. In so doing, it is expected to contribute to the efforts of the three levels of Brazilian governance in favor of a more efficient performance of state and private companies in the management of integrated sectors of fresh water supply, sewage, and the universalization of such services until 2030. The current study can be used to support managers in their decisions considering the efficiency measurement in order to reduce potential wastes and maximize the service coverage and the operational results.

This article is structured in four sections, including this introduction, presenting the main works that use DEA in the Brazilian basic sanitation sector that are close to the objectives of this study. The second section describes the data set employed and the DEA empirical model based on the multiple optimization method, detailing the estimation parameters to assess the efficiency of Brazilian basic sanitation. Section 3 presents a realistic view of the challenges faced in the operational results and coverage of BSU services, given the current levels of infrastructure, human and financial resources in the sector. In addition, it brings discussions about BSU performance and potential efficiency gains. Finally, Section 4 presents the final considerations of the challenges and evidence found for the integrated management of water supply and sanitation.

### Efficiency Measurement in the Sanitation Sector

The measurement of efficiency aims to evaluate productivity through a study of the relationship between resources and products that are part of the production function [36,37]. The conventional DEA model is an approach widely adopted by many studies in different sectors, e.g., education, energy, health, sanitation, finance, telecommunications, and in different countries [9,29,38,39]. Specifically on the sanitation sector, Abbott and Cohen [28] highlight that, in the last two decades, studies on efficiency have been carried out using mostly the conventional DEA method. Berg and Marques [27] follow this observation, extending the analysis of 190 quantitative studies associated with the performance evaluation of the basic sanitation sector and that the main techniques are concentrated on the application of conventional DEA. Thus, the works reviewed in this section have in common the use of the DEA approach and because they have dedicated themselves to measuring the efficiency of water supply and sewage services. Four main characteristics are focused on: application of conventional DEA; application to national data sets; main selected variables; and model specification parameters.

To summarize the assessment of efficiency in water use and sewage collection, Table 1 shows the resources and products used in the different surveys applied to Brazil and their respective studied periods, numbers of BSUs in the sample, territorial coverage (TC), model orientation (MO), scale yield (SY) and efficiency scores measured by DEA (EFF). In general, the efficiency assessment models consider the integration of the two dimensions, water and sewage, and normally consider, as resources, operating exploration expenses (OEE), own labor force (OLF) and the available infrastructure of the extension of the general water (IEW) and sewage (IES) network and, as products, direct operating revenues (DOR), the active connections system in the general water (ACW) and sewage (ACS) network, the total volume of treated water (VTW), the total volume of treated sewage (VTS), and the demand of the population served with water (PSW) and sewage (PSS) services.

Regarding to the study’s scope criterion, sector studies have macro scope [16], with a comparison of efficiency between countries as being BSU, and micro [24], with emphasis on performance of specific local companies or BSU based on aggregate state and regional results. Another criterion that varies in studies in the sector concerns the format of the efficiency frontier of the DEA model. In addition, there are also different types of guidance: input (input maximization) or output (product maximization), as shown in Table 1, about the model orientation (MO).

Another limitation of the studies carried out on efficiency is the reduced number of decision-making units adopted. Previous studies disregard the provision and result of the basic sanitation service at the municipal level, by a company that is responsible for the sanitation of the municipality, even though it is a subsidiary of a state company. The situation of limitation of the decision-making unit (decision-making unit—DMU) in the sector’s efficiency studies causes in the DEA a high concentration of DMU considered as benchmarks. Another criterion of inconsistency concerns the use of the variables of revenues and operational expenses of the basic sanitation sector. It should be noted that the financial variables of the Brazilian basic sanitation sector are determined by the federal government in a unified manner, based on the total invoiced revenue for water plus sewage, as well as the expenses resulting from these services. Thus, no distinction is made between water and sewage in the OEE and DOR variables. Such a peculiarity must be observed for the other input and output variables when dealing with water and sewage integrated management. In other studies, the efficiency estimation model does not standardize the calculation of the other variables.

Therefore, DEA efficiency measurement works in the basic sanitation sector are far from being scientifically uniform regarding the criteria for choosing input and output variables, in addition to the different research scope, model parameters, sample representativeness and methods of using the financial variables of BSU operating income and expenses. According to Table 1, the efficiency indices, on average, were 73.5%, however, the amplitude (0.9748–0.3993) of results was 57.6% points and the standard deviation was 23.2%.

The results of the DEA model are sensitive to the choice of resources and products, so that an arbitrary choice is potentially generator of biases that are difficult to identify regarding the calculated value of efficiency. In view of this, one of the limitations of the highlighted studies is the arbitrary selection of the input (resources) and output (products) variables, a fact that alone makes it difficult to compare the results between different studies, as shown in Table 1. In this direction, for example, Macedo [18] uses different inputs and outputs than Motta and Moreira [31], so that the average efficiency varied from 84.7% to 43.1%. The work by Ortega et al. [16] presents the lowest value and Carmo and Távora-Junior [32] the highest value, confirming the variability of the results according to the model specifications.

Thus, it is not easy to evaluate the relative performance of an organization, comparing the performance to a partner of excellence (benchmark), when there are multiple inputs and multiple products to be considered in the analysis of the production system. The difficulties increase when the relations between inputs and products are complex and involve unknown balances. Therefore, this study evaluates performance with an M-DEA structure that aims to reduce the arbitrariness of the choices of inputs and outputs in the process of optimization and performance comparison between the BSU of the Brazilian sector.

## 2. Materials and Methods

**Figure 1:**
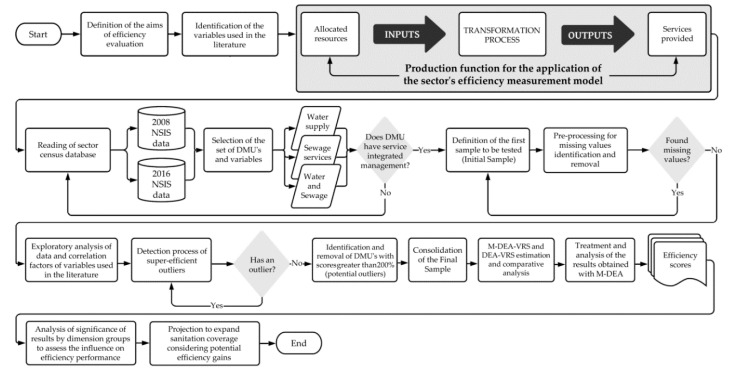
Flowchart of the approach adopted in applying the M-DEA. Source: Author’s own elaboration based on Golany and Roll [40] and Slack et al. [41].

This section is divided into four parts. The first presents the principles of M-DEA and an overview of the main stages (2.1). Then, how to obtain the efficiency measures and the variables used in the model (2.2), the significance analysis (2.3) and the data used (2.4) are described. Figure 1 shows all the steps followed in the evaluation of technical efficiency in the case of the present study.

In summary, the main steps followed and illustrated in Figure 1 to achieve the objectives were: to identify the input and output indicators commonly adopted in the literature; control the detection of outliers in obtaining efficiency; perform multiple DEA executions, considering all possible combinations of inputs and outputs; obtain the total efficiency of each DMU by means of the arithmetic mean of the efficiency scores of all possible combinations between inputs and outputs, considered in the efficiency model; assess whether efficiency is influenced by factors for aggregating results (dimensions); and estimate the potential level of expansion of basic sanitation coverage, based on the simulation of potential efficiency gains in Brazilian basic sanitation companies.

### 2.1. Introduction and Principles of the M-DEA

This study uses the M-DEA approach to calculate the efficiency index of the basic sanitation sector in Brazil. It is an extension of the conventional DEA model combined with the formulation proposed by Stosic and Fittipaldi [35]. Among the characteristics that make DEA an operational model of great interest in the literature for evaluating DMU performance and for guiding of decisions strategic institutions and companies, we can mention: it does not require the determination of functional relationships between inputs and outputs (nonparametric modeling); it is not restricted to single and singular measures of inputs and products; it allows the use of discretionary variables with a multidimensional perspective; it defines relative competitive positioning of a set of DMU in a synthetic way in a single indicator. Therefore, the level of efficiency is calculated by interactions between multiple inputs and outputs in a production relationship, making it possible to identify better standards of excellence (benchmarks). Thus, the estimated production ratio can be synthesized by: $Y\left(y_{1},y_{2},\ldots ,y_{M}\right)=f\left(x_{1},x_{2},\ldots ,x_{N}\right)$, where $y=\left(y_{1},y_{2},\ldots ,\;y_{M}\right)$ is the product vector and $x=\left(x_{1},\;x_{2},\ldots ,\;x_{N}\right)$ is the input vector. To mitigate the lack of a standard on which inputs and outputs should be used in the model, the M-DEA allows the repetition of the iterative execution of the calculation, estimating the efficiency of each DMU from all possible combinations of input and output variables used.

The efficiency of each DMU is assessed by a score, called technical efficiency (TE), which varies between 0 (lowest level of efficiency) and 1 (highest level of efficiency). For the calculation of TE, there are two basic assumptions for building the efficiency frontier. In the model assuming constant returns to scale (constant returns to scale—CRS), the efficiency frontier is given by a linear production function defined by the DMU with higher relative productivity, under the assumption that all units operate at an optimum scale of production and that the increase in production is proportional to the increase in input because they are homogeneous DMUs [42]. On the other hand, in the model with variable returns to scale (variable returns to scale—VRS) the efficiency frontier has a concave format, admitting in this case that the DMUs under analysis can operate under different production scales, being a more realistic model when considering the existence of this heterogeneity in the production process of units with different sizes and complexities [43].

It is noteworthy that the M-DEA is an extension of the DEA model with multiple executions of the conventional model (CRS, VRS and other variants), considering all possible combinations of inputs and outputs to calculate the efficiency scores for a given DMU. Equation (1) shows the conventional DEA model under the hypothesis of VRS:

(1) $$\underset{\theta ,\lambda }{\mathrm{Max}}\theta \;\mathrm{Subject}\;\mathrm{to}:\;x_{i0}-\overset{n}{\underset{k=1}{\sum }}x_{ik}\lambda_{k}\geq 0\;\forall_{i}\;\overset{n}{\underset{k=1}{\sum }}y_{mk}\lambda_{k}-\theta y_{m0}\geq 0\;\forall_{m}\;\overset{n}{\underset{k=1}{\sum }}\lambda_{k}=1\;\lambda_{k}\geq 0\;,$$

where y represents the output, x is the input, λ is a vector of weights, $\sum_{k=1}^{n}\lambda_{k}=1$ is the convexity constraint admitting VRS, θ is a scalar indicator of TE. This model determines efficiency by optimizing the division between the weighted sum of outputs (products) and the weighted sum of inputs (resources), selecting sets of optimal weights for each DMU. In practical terms, its combination with the M-DEA method allows the iterative calculation of the DEA execution for different possible choices of subsets of input and output variables.

**Figure 2:**
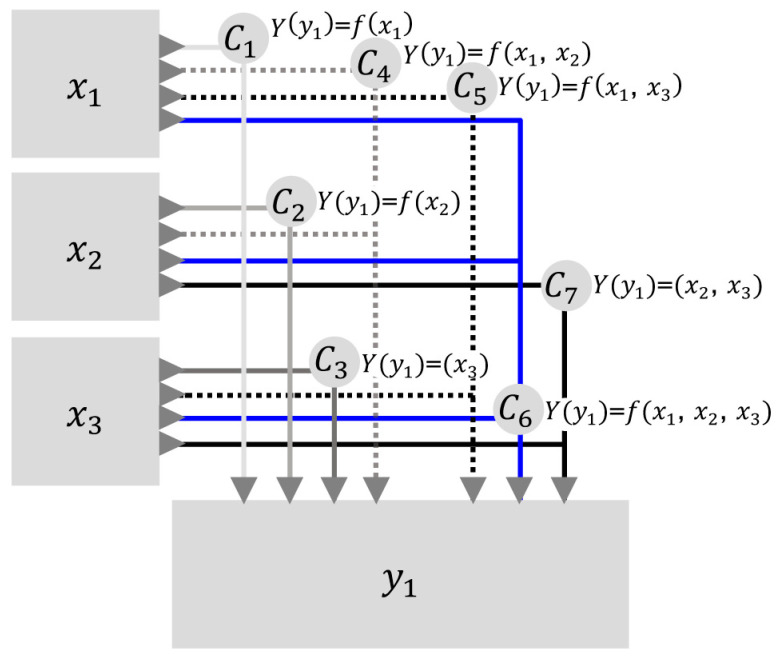
Graphical representation of the M-DEA model from the theoretical formulation of Stosic and Fittipaldi [35]. Source: Author’s own elaboration based on Stosic and Fittipaldi [35].

Thus, when considering the multiple combinations of inputs and products, M-DEA avoids the bias of biased selection of variables and, therefore, performance estimates are obtained that are less sensitive to variations in the production set within the respective efficiency frontiers of the DMU [44,45]. Following the approach of Stosic and Fittipaldi [35], the relevant inputs (n) and products (m) are considered, where n∈{1,2,…,N} inputs and m∈{1,2,…,M} outputs are combined in order to guarantee the maximum number of input-output combinations, given by $N_{c}=\;\left(2^{N}-1\right)\;\left(2^{M}-1\right)$. The DEA is then performed for all DMUs with each combination of input and output variables. Figure 2 explains a basic model, as an example, with three inputs and one output for the execution of the DEA illustrating the multiple combinations of this case ($N_{c}=7$) for the iterative calculation of the efficiency score by the M-DEA [35].

The total efficiency for a given DMU is calculated using the arithmetic mean of the efficiency scores of all seven possible combinations, despite the achievement of the efficiency score in single stage by the conventional DEA [43], which would occur with the estimation of efficiency through the variables observed in node $C_{6}$ of Figure 2. Thus, DMUs are evaluated in all different possible contexts (combinations of input and output variables) and the effects of arbitrariness in choices are reduced of the inputs and outputs of the efficiency measurement model of the Brazilian basic sanitation sector. In this way, this work differs from the art state in that it uses an extension of the DEA model with multiple combinations of results (M-DEA) to calculate the non-biased sectoral technical efficiency (TE) resulting from all combinations between inputs and outputs considered in the efficiency model. The advantage of this model is that it reduces the arbitrariness of the choice of variables in the estimation of technical efficiency and improves the discriminatory power of the DEA model [35,44,45]. To address this issue, M-DEA invests in significant computational effort to process a large number of inputs and outputs, and their respective combinations.

### 2.2. Efficiency Model

This score is assessed in the two years investigated, 2008 and 2016, according to data available after the regulatory framework of the sector [46], to observe trends in changes in efficiency levels. In Brazil, the infrastructure and water and sewage in a municipality is usually provided by local agencies, even though they are part of state companies. In this research, the BSU or DMU is the municipal sanitation agency that provides infrastructure and water and sewage services.

The analysis developed in this study used the product-oriented M-DEA model, which focuses on maximizing results from the inputs used. This guiding principle allows verifying the maximum quantity that can be produced, given the fixed quantity of inputs. Considering that 85% of the total number of companies in the period is public, it is worth noting that there are some legal restrictions for layoffs of tendered labor and to flexibly tie wages to the level of labor productivity, factors that, in general, impose more rigidity to the inputs of public companies in the country. This characteristic also reflects in the census data of the sector, which considers specifically and exclusively as information about the human resources of the BSU the amount of own labor force (company employees).

The results consist of an average of the 105 efficiencies calculated for each DMU, considering all the possibilities of choosing variables used as inputs and products. For processing the M-DEA model, an algorithm was implemented in the R language (version 3.5.1, R Foundation for Statistical Computing, Vienna, Austria). Table 1 initially showed four (4) input and seven (7) output variables according to the frequency of use in the literature, which aims to evaluate the efficiency of water and sewage provision, considering the availability of information, recurrence and the integrated sanitation management logic. The official databases of the National Sanitation Information System (NSIS), however, unifies the financial results of water and sewage in the variables of revenue (DOR) and operating expenses *(*OEE). With this in mind, the present work groups the water and sewage variables, emphasizing the focus on integrated management of basic sanitation and reducing the dimension of entry and exit. In order to standardize the concept of integrated management, the non-financial variables used in M-DEA are also unified.

Given the heterogeneity of the operating sizes of the different BSUs under analysis, the enveloped model used in this research admits variable returns to scale [43] in order to ensure a better evaluation of efficiency. The set of Brazilian BSU has varying sizes of service provision scale, whether it is represented by the number of jobs they generate, the size of their assets, the potential of their consumer market, revenue, the extent of their network and infrastructure, etc., in such a way that the basic sanitation sector’s BSUs tend to have different scale returns.

Table 2 illustrates the variables of the system of indexes of entry and exit of the TE model from the public database of NSIS, which consolidates the official data of the sanitation sector in Brazil. It is a consistent source for the objectives of this work. Alongside this, it is opportune to explain that the declaration of information by BSU in the NSIS database is a normative obligation and is public information, which is available in open data format.

The final model consists of three input variables, which include: financial information on operating exploration expenses (OEE), own labor force (OLF), and available infrastructure, the latter resulting from the combined values of IEW + IES (extension of general network). In the output of the M-DEA model, four variables are used for the analysis of product maximization, which considers: financial information on direct operating revenues (DOR) plus variables for capturing operating results, which were aggregated and converted into a single variable of water plus sewage, ACW + ACS (total active connections), VTW + VTS (total treated volume) and PSW +PSS (total population served), as shown in Table 2. The financial variables for 2008 were corrected by the IPCA-IBGE inflation index, referring to the Broad National Consumer Price Index, which measures the increase in the values of tariffs for public services [48]. The cumulative factor of the reference index for the period up to 2016 was considered for the purpose of updating the values of variables DOR and OEE of 2008. Thus, according to the definition of N=3 inputs and M=4 outputs, a total of $N_{c}=\;\left(8-1\right)\left(16-1\right)=105$ possible combinations of input-product was obtained for each DMU in the sample.

#### Projection of Potential Efficiency Gains

Once the TE scores for each BSU are calculated, it is possible to estimate the potential level of expansion of basic sanitation coverage in Brazil, by geographic region and by federation unit. This is especially important to overcome the slow sectoral progress and the historical absence of good performance measures to achieve the universalization of basic sanitation through the individual increase in the production of Brazilian BSUs. For the simulation of the potential level of the optimal coverage tax of a given geographic region, the most recent survey data for the year 2016 is used.

The projected service rate in the j-th geographic region ($PS_{j}$), based on the parameters from the M-DEA model, is calculated by:

(2) $$PS_{j}=\frac{\sum_{i=1}^{I}\mu_{i}^{*}}{tp_{j}}\leq 1$$

where $\mu_{i}^{*}$ is the projected value of the population served by the i-th BSU and $tp_{j}$ is the total population of region j, therefore $PS_{j}\leq 1$. The projected value $\mu_{i}^{*}$, in turn, is obtained by $\mu_{i}^{*}=\left(\mu_{i}/TE16_{i}\right)$, with $TE16_{i}$ the efficiency score in 2016 and $\mu_{i}$, the current number of people served with piped water and sanitation. If a BSU has a TE=0.60, for example, the projected value of the population served would be $\mu_{i}^{*}=1.67\mu_{i}$, expanding the outputs given the input levels, to reach a maximum level of efficiency.

### 2.3. Significance Analysis

In order to assess whether efficiency is influenced by (i) the administrative nature of the company, (ii) the geographic region, (iii) the population range it serves, (iv) the scope of operations, (v) the size of the company, and (vi) the infant mortality rate at the place of operation, the TE was calculated in six different strata (grouping dimensions). The Mann-Whitney non-parametric test was used to assess differences in performance, considering a significance level of 5% [49]. For this, each grouping dimension was summarized at the end in two groups, to verify whether the $\bar{TE}$ scores in Group 1 (G1) are higher than those in Group 2 (G2), where $H_{0}:$ the two groups are equal versus $H_{1}:$ the two groups are not equal. The adherence dimensions, that is, hypotheses to be confirmed, were six: D1 = BSU public administrative nature vs. BSU private administrative; D2 = BSU Northeast region vs. BSU other regions of Brazil; D3 = BSU operation in municipalities range greater than 100 thousand inhabitants vs. BSU operation in municipalities range less than 100,000 inhabitants; D4 = BSU with local/municipal management vs. BSU with regional/state management; D5 = BSU size with more than 500 employees vs. BSU size with less than 500 employees, and D6 = BSU operation in a municipality with low infant mortality vs. BSU operation in municipalities with higher infant mortality.

### 2.4. Data

This section analyzes the data used in the M-DEA model to calculate the TE of the basic sanitation sector in Brazil, according to the definition of the technical efficiency of the integrated management of water supply and sewage described above. In addition, it demonstrates the exploratory analysis of the data and explains the specification of the outlier detection method to mitigate possible problems in the efficiency estimation.

In this study, data from the NSIS are used, which provide official national information about the BSU and its operating income, expenses and productivity results. The reference years for estimating efficiency are the basis of NSIS [47] and NSIS [8], serving to examine the dynamics of the basic sanitation sector in Brazil regarding the sector’s productivity based on the national regulatory framework. It is worth highlighting that the initial year of the database was marked by the first consolidated assessment of the diagnosis of water and sewage services in Brazil after the regulatory framework of the sector came into effect [46]. The most recent year was chosen due to the availability of the database with the official agency of the federal government responsible for collecting census information on basic sanitation in Brazil.

**Figure 3:**
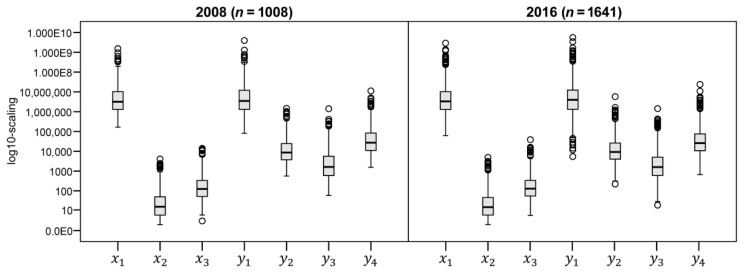
Boxplot of input and output variables, 2008 and 2016.

The initial sample consists of 4610 BSU in 2008 and 5687 BSU in 2016. In Brazil, the provision of the water supply and sewage service can occur in a segregated manner, a company responsible for each service, or in an integrated manner, only one company for both. For the present work, the final sample considers public and private BSUs with integrated operation in the water supply and sewage treatment sectors. In this way, the sample that contemplates integrated operation had 1464 BSU in 2008 and 2062 BSU in 2016. The exploratory analysis of the input and output data used in the M-DEA can be seen in Figure 3, highlighting the atypical points (○) and the extreme atypical points (*), using the boxplot limits (Upper Limit (UL): °°°Q3 + 1.5 IQR; * UL: Q3 + 3 IQR; Lower Bound (LB): °°°Q1—1.5 IQR; * LB: Q1—3 IQR), where Q1 is first quartile (P25), Q3 is the third quartile (P75) and IQR is the amplitude of the interquartile range (P75—P25).

Figure 3 shows the boxplot of the input and output indicators of the data collected for the years 2008 and 2016. Table A2, in the Appendix A, lists the descriptive statistics of the minimum, maximum, average and standard deviation values. The sample includes basic sanitation companies that vary in size and coverage, so the boxplot shows the importance of caring for outliers in data analysis for M-DEA modeling.

As an example, the values of $y_{4}$ show that Brazil is composed of municipalities with different demographic densities. The existence of variable returns to scale due to the great variation in the sizes of companies, measured by their assets (e.g., $y_{1}$ and $y_{2}$, respectively, revenue and active connections) corroborates the specification of the wrap model adopted in this study. Note that the set of variables analyzed is commonly adopted in the sector’s literature, as shown in Table 1, and that in the present study the use of M-DEA aims to measure technical efficiency in the various possible combinations between inputs and outputs. With this, all the subsets different from $x_{1}$, $x_{2}$, $x_{3}$ and $y_{1}$, $y_{2}$, $y_{3}$ and $y_{4}$ are successively chosen for all DMU’s, thus avoiding arbitrariness in this choice. Based on the correlation matrix in Table A3, in the Appendix A, all variables have significant positive correlations, above 0.79 in 2008 and above 0.60 in 2016. Like Wang et al. [50] and also following the protocol of Dyson et al. [51] on correlated factors and the pitfall of omitting variables in the context of measuring efficiency, we kept all resources and products variables.

Considering the potential that the atypical points shown in the boxplot represent noise-causing observations in the estimation of the TE, we proceeded next to what the literature considers as outlier detection control. The outlier detection and removal procedure was adopted based on Banker and Chang’s model [52]. As a parameter, levels greater than 200% were considered in the estimation and re-estimation, being removed from the final sample and reported in Table A5, in the Appendix A, all the observations identified under the precepts pointed out in the study of Banker and Chang [52]. This approach is based on the concept of super-efficiency in which a DMU can achieve unrestricted scores, ranging from zero to more infinite, to detect errors or discrepant observations in the database. In the present study, the final adjusted sample totals 1628 observations for 2016 and 1001 observations for 2008.

Then, after the elimination of DMUs with a super efficiency greater than 200%, the M-DEA model was applied to the sample without the presence of outlier units whose results are presented in the following section. It is worth mentioning, beside this, the existence in the literature of other techniques for detecting and removing outliers, such as the one developed by Simar [53], including the order-m frontier, and Kapelko and Stefanou [54], who apply the method adapted from statistics when analyzing the ratios of output to input in determining outliers.

## 3. Results and Discussion

In this section, a comparison between the efficiency results obtained in the DEA and M-DEA models is initially presented. Then, we proceed with the demonstration of the results and analysis of efficiency based on the M-DEA model.

### 3.1. Comparison of Results between DEA and M-DEA

**Figure 4:**
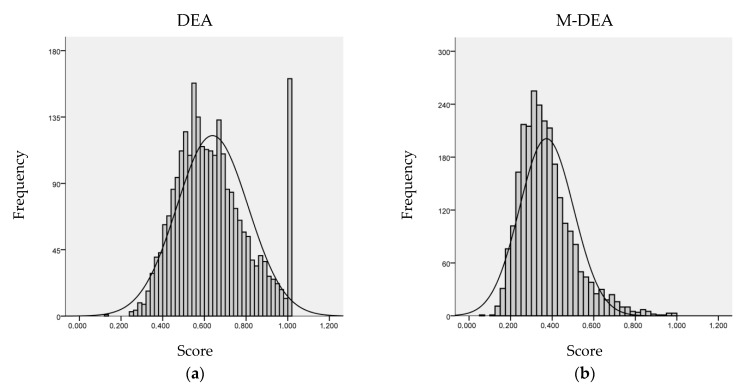
Frequency distribution of the efficiency index of the DEA (**a**) and M-DEA (**b**) models without outliers for 2008 and 2016.

To present the estimates calculated by the M-DEA-VRS, the results were compared with the conventional DEA-VRS. Figure 4 compares the frequency distribution of the efficiency index calculated in the conventional and multiple wrap models, for the periods of 2008 and 2016. Therefore, it is worth noting that the production function for the efficiency frontier was built based on the sample of each year, to then measure the TE of the basic sanitation units using DEA and M-DEA. It is possible to notice in the result of the DEA model the existence of an excessive amount of efficient BSU in the right tail and, also, a low heterogeneity of performance in the frequency distribution, resulting from the unique estimation of the DEA based on the selection of the input and output variables, as discussed in Section 2. This type of classic DEA approach can favor a particular BSU group as shown in Figure 4. The distribution of the M-DEA model, on the other hand, avoids problems of high concentration of efficient BSU as benchmarks, situated in the distribution of efficiency scores in the tail of the positive extreme towards 1, correcting the potential selection bias of the variables used.

The demonstration of the results of conventional DEA (Figure 4a) and M-DEA (Figure 4b) at this point is to show that, while a researcher can argue the use of a set of inputs and outputs to estimate the technical efficiency of the sector, this can increase the failure of the power of discrimination of the DEA and cause the saturation of efficiency in the classification of DMUs of the observed sample. Similar behavior is observed in the empirical results obtained with M-DEA by Fernandes and Sousa [45] and Lima e Silva et al. [44].

Therefore, the M-DEA was proposed to mitigate the problem of lack of uniformity in the variables adopted in the sector’s work. As the M-DEA is based on multiple combinations of input and outputs, the result consists of an average of the efficiencies calculated for the TE of each BSU, considering all the possibilities of choosing variables used as inputs and products. It should be emphasized that the M-DEA estimates for the calculation of the TE become more appropriate in the present analysis since all DMUs have the same chance of being evaluated in all different possible contexts, as well as due to the lack of uniformity in the variables used in the studies on the efficiency of the sector, according to Abbott and Cohen [28] and Berg and Marques [27].

The DEA analysis has an average efficiency score of 0.639, with a standard deviation of 0.171, for 2629 observations, in the aggregate of the two periods evaluated. Concerning the M-DEA, there is an average efficiency score of 0.373, with a standard deviation of 0.131, for the same number of observations. In the M-DEA, not BSU reached the maximum level of efficiency, while in the DEA 161 BSU reached the maximum level. The DEA estimate is skewed upwards and shows that the BSU ratings have an overestimated magnitude with much higher efficiency values for the basic sanitation sector than the results of the multiple models. Thus, the estimates using the M-DEA correct potential of arbitrary parameterization in the selection of variables, being the reference model for the other analyzes in this article.

**Figure 5:**
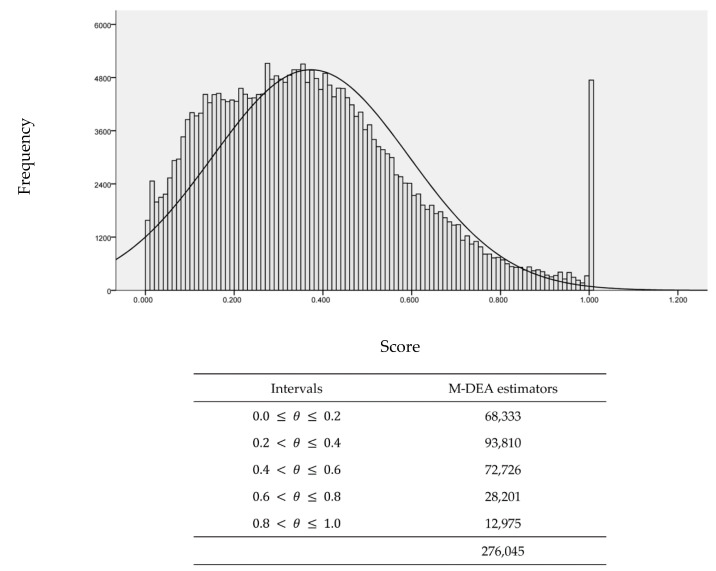
M-DEA estimators (*n* = 276,045) and summary by class intervals of scores.

Figure 5 shows the statistical distribution of efficiency measures (θ) for all 105 possible combinations of resources and products for each DMU in the sample (*n* = 2629), with 276,045 optimization problems solved. The M-DEA assumes that the DMU is efficient when it presents in all scenarios of combinations of input and output the best relative productivity, that is, the best score not only in one combination, but in the 105 possible combinations in the case of the present study.

It must be understood that each DMU has a competitive position according to the set of production possibilities. This is relevant to demonstrate that the absence and/or addition of a variable can affect the production possibility frontier. The conventional DEA model is sensitive to the variable selection criteria, so it can cause bias when estimating technical efficiency measures (Figure 4a). The M-DEA contemplates different combinations of input and output variables, reducing the effects of arbitrariness in the choices of resources and products of the model for measuring the efficiency of the basic sanitation sector, as shown in Figure 5.

It is highlighted that in Figure 4b the M-DEA shows the average distribution of 105 possibilities for each DMU, while the DEA shows only a combination (Figure 4a). For this reason, a difference is expected between the DEA and M-DEA distributions, which does not characterize the method’s lack of robustness. It is emphasized that the M-DEA originates from the conventional DEA model itself, being in a multidimensional perspective and considering a varied range of situations. In the present case, it considers 105 contexts, as Figure 5 illustrates. As previously highlighted, there is no single specification in the models that assess the efficiency of basic sanitation, which makes the direct comparability of the results unfeasible. Just as there is a problem in the DEA related to small sample variations [36,37], the same problem occurs with the selection of variables [51] and the M-DEA tries to mitigate this second issue. In this sense, the TE generated by the M-DEA and detailed in the following section contributes to the analysis and evaluation of competitive positioning and to guide strategic decisions in the Brazilian basic sanitation sector.

### 3.2. Efficiency Analysis Based on the M-DEA Model

Table 3 lists the results of TE scores by BSU administrative network using the M-DEA model. Columns TE08 and TE16 show the efficiency in the sampling period, and the Δ% column shows the difference in performance between the two periods analyzed. In addition, results are also stratified by geographic region, population range, administrative scope, size of the company, and ranges of infant mortality rates at the place where the BSUs operate.

The average TE in Brazil reveals that the integrated management of basic sanitation in Brazilian municipalities has remained inefficient over time and far from its ideal value (TE=1). For example, the average value of ${\bar{TE}}_{2016}=0.3903$ points out that the coverage of service to the Brazilian population can increase by about 61% when all the BSUs in the base year 2016 simultaneously improve efficiency without changing the current levels of infrastructure, human, and financial resources of the sector. Although there are margins to reduce waste and regional distortions, there is an increase of 13.4% in the TE indices between 2008 and 2016.

Dividing the BSUs managed by the public administration and the private administration, the results found demonstrate that efficiency between 2008 and 2016 increased by 16.0% in private BSUs and 12.8% in public BSUs. It is important to highlight that in Brazil, 85% of the BSU are publicly managed and only 15.1% are privately managed, which accounted for 15.8% of the total investment in the sector in 2016 [8].

In regional terms, the TE, within 2008–2016, pointed to a greater advance for the Southeast regions, followed by the South, Midwest and North regions of Brazil. Despite the lowest percentage of growth, the Northeast region, one of the poorest and least developed among all [7], presented the best performance throughout the analyzed period. In the northeast, the highest concentration of BSU with integrated management occurs in the most populated areas. Table 3 shows that population size is a significant factor in explaining the TE index, as efficiency tends to increase with the size of the municipality. In general, municipalities with less than 100,000 inhabitants tend to have small businesses. Such a result confirms that the performance of the provision of water and sewage services is influenced by the location, and can be framed as a representation of the great technical and socioeconomic inequalities faced in the Brazilian regions.

### 3.3. Significance Analysis

In order to determine whether there is statistical evidence that the efficiencies found are significantly different due to aggregation factors of the results (dimensions), a Mann–Whitney test was performed to analyze the trend of efficiency results in the following dimensions: administrative, public or private nature (D1); northeast region in relation to other regions of Brazil (D2); population greater than 100 thousand inhabitants in relation to the other population groups (D3); scope of action with local/municipal management compared to regional/state (D4); company size, large compared to the others, with less than 500 employees (D5); operation in a city with low infant mortality compared to cities with medium and high infant mortality (D6). Table 4 shows the summarization of the central trend of the difference in efficiency values by dimension in 2008 and 2016.

Table 4 shows that the size of the company and the population range indicate significantly higher levels of TE, respectively, D5 and D3. In this sense, the TE results suggest gains in scale: the larger the size of the company and the population density of the municipality in which it operates, the higher the efficiency score of the sanitation company operating in the region. Table 4 also shows that the other dimensions have a low effect on the result of TE, despite indicating statistical significance at 5%. However, for the 2016 D1 results, the TE value indicates, for example, that the BSU is of a public or private nature does not have a significant increase or decrease in TE (p > 0.05).

### 3.4. Public Policy Implications

**Figure 6:**
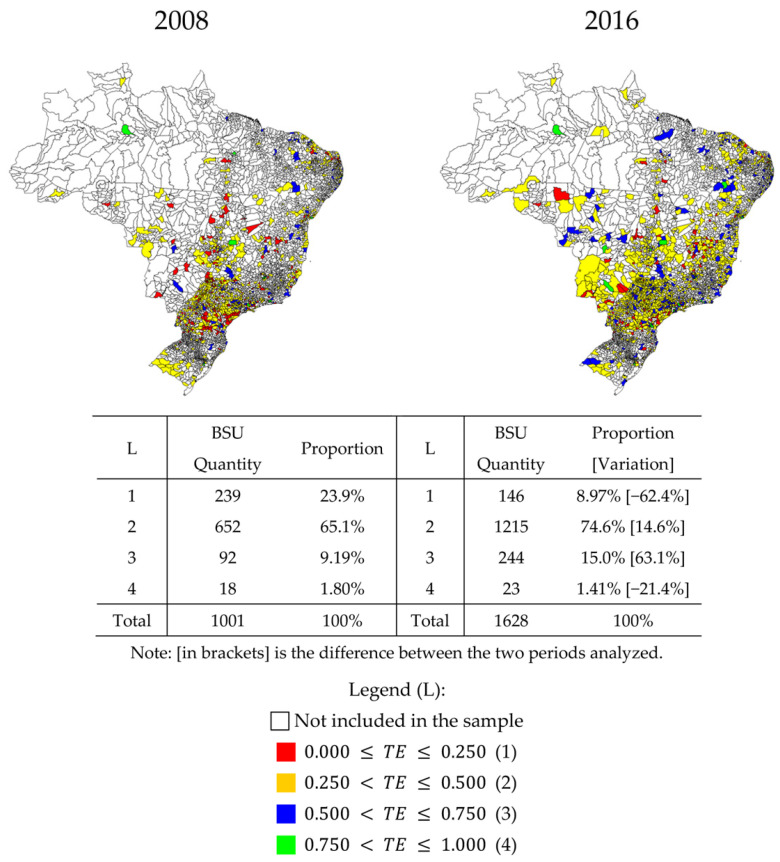
Spatial distribution of sectoral technical efficiency (TE) for basic sanitation management in Brazil. Source: Author’s own elaboration based on the research result.

This study provides contributions to the literature in the area with implications for the formulation of public and managerial policies aimed at setting goals and designing managerial incentives in the Brazilian basic sanitation sector. The efficiency estimate deepens the understanding of the reality of the natural and economic resources allocated in various regions of the country from the regulatory framework of the sector [46]. Figure 6 shows the distribution of TE by municipality in Brazil. It is noted that the global efficiency scores in the integrated management of basic sanitation in Brazilian municipalities are predominantly below TE ≤ 0.500 (83.6% of the BSU have this performance in 2016), with recurrence of inefficiency in different regions of the country. This focuses on the existing challenge of the low level of performance seen in the red and yellow areas inside the map. Figure 6 shows the persistent problems of productivity and sanitation management in the BSU for the entire sample period, so that the population of these municipalities should have greater coverage of water and sewage services. The blank areas on the map correspond to municipalities that do not have integrated management of basic sanitation in the years of 2008 and of 2016; therefore, they did not compose the final sample of this study.

From the results presented in Figure 6, only 18 sanitation companies in 2008 and 23 companies in 2016 had a high level of efficiency, with 0.750 < TE ≤ 1.000. Given the high concentration of points on the map, it is not possible to view these municipalities, which are located in 2008 in Belo Horizonte/MG, Brasília/DF, Salvador/BA, Manaus/AM, Fortaleza/CE, Nova Iguaçu/RJ, Curitiba/PR, Contagem/MG, Recife/PE, São José do Rio Preto/SP, São José dos Campos/SP, Imperatriz/MA, Betim/MG, Cabedelo/PB, Duque de Caxias/RJ, Balneário Camboriú/SC, Porto Alegre/RS and Ribeirão das Neves/MG.; and in 2016 in Salvador/BA, Brasília/DF, Belo Horizonte/MG, São Gonçalo/RJ, Duque de Caxias/RJ, Abreu e Lima/PE, Mesquita/RJ, Guarulhos/SP, Tupandi/RS, Belford Roxo/RJ, São Bernardo do Campo/SP, São José dos Campos/SP, Petrolina/PE, Curitiba/PR, Campo Grande/MS, João Pessoa/PB, Manaus/AM, Goiânia/GO, Colombo/PR, Rondonópolis/MT, São José dos Pinhais/PR, Paulista/PE and Porto Alegre/RS. It is important to highlight, for example, that in 2016 1.41% (23) of Brazilian companies were observed in the category 4-high level of efficiency, 15.0% (244) in the category 3-medium level of efficiency, 74.6% (1215) in the category 2-low level of efficiency and 8.97% (146) in the category 1-very low level of efficiency, which can help in guiding public policies on sanitation management in Brazil.

In addition, more than half of the 1628 BSU operating in 2016 did not reach the national average of efficiency (TE16=0.3903) in water management and sanitation. A similar situation was found in 2008. This geographic distribution of TE points out that the level of efficiency can increase on a large scale and supports decision-making on actions to better distribute the available resources in order to improve the overall performance of the sector as a whole.

The BSUs in Brazil need to continually learn to position themselves strategically in the sector, matching the best efficiency standards of their respective geographic regions. Table 5 presents the benchmarks by region in 2016, which can be used to strengthen regional management of natural and economic resources, as well as technological innovation in the sector. BSU EMBASA, in Salvador/BA, is the most efficient in the country, in contrast to the company PMCNP, in the municipality of Campos Novos Paulista/SP, which presented the worst national performance in 2016. The most efficient BSU operates in a municipality classified in the band with more than 100 thousand inhabitants (population of 2.9 million inhabitants). The less efficient BSU operates in a municipality classified below 10,000 inhabitants (population of 4870 inhabitants). The BSU-benchmark is managed by the public administration and has a state/regional administrative scope, managing a group of 365 municipalities in the state of Bahia. The least efficient is managed by the public administration and has local/municipal administrative scope, with a legal nature of direct public administration. It is necessary to direct efforts to control inputs, overcome technical-operational problems and improvements in management technology for planning and implementing the necessary changes, such as reducing operating costs, pricing/tariff strategies, introducing technological innovations to mitigate losses in water distribution, influence on the regulatory and market structure and constant assessment of the competitive position. These considerations are important to demonstrate that there is a set of options for a BSU to produce the same product more competitively and, therefore, with a greater range of services. Such decisions presuppose investments in improving production processes/new technologies.

The systematic comparison of performance with benchmark companies in the sector, like the company EMBASA/BA, the best performance of 2016 (BR), assumes a relevant space to define standards to measure performance and incorporate into the sector’s regulatory framework, as shown in Table A4 in the Appendix A. A comparison of the company’s resources and products with the highest TE value (benchmark) and the lowest TE value is shown in Table A4. The results have important managerial implications for less efficient BSUs, indicating, for example, the extent to which they lag behind the best performers in the industry, and allowing them to identify BSUs that have more modern and consolidated business management systems. This finding is clearer when analyzing, for example, the values of the variables of operating expenses and the direct operating revenues of inefficient BSU. As an example, BSU in Campos Novos Paulista/SP (lower TE16) operates with a 97% revenue deficit, see Table A4, y1 vs. x1. This same problem is reflected in 1/3 of Brazilian BSUs that operate with lower revenues than expenses, signaling economic unfeasibility and management quality problems in the sector [47]. In this direction, the annual operating cost per active connections on the network of the benchmark company is R$ 1308.90 and, in the less efficient company, it is R $ 3445.95, which has a cost 2.63 times higher than that. The benchmark company operates with a revenue margin of over 62%, while the other has 97% of operating loss. The most efficient company is classified in the category 4-high level of efficiency (0.750 < TE ≤ 1.000). The list of the 20 highest and lowest TE is found in Table A1, in the Appendix A.

Considering the orientation towards production, the aim of this study was to maximize production by fixing the resources (inputs) used to provide services. The indicators developed in this article for the year 2016 (TE16) made it possible to project an expansion of the coverage of basic sanitation, correcting all technical inefficiencies of BSU. Even though it is a hypothetical scenario, this assessment allows to measure the impacts of waste reduction on the number of people served with basic sanitation if the BSU used the resources correctly.

**Figure 7:**
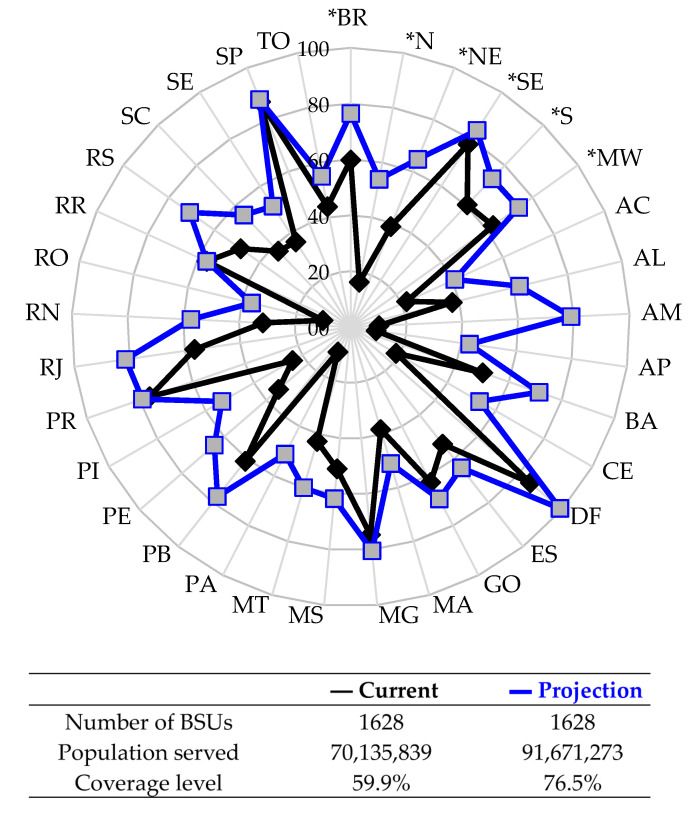
Percentage of the population served with water and sewage, considering the estimated efficiency gains within the final sample in relation to the current level of efficiency. Results by federative units in Brazil and by Brazilian regions (*): BR = Brazil; N = North Region; NE = Northeast Region; SE = Southeast Region; S = South Region; MW = Midwest Region. Source: Author’s own elaboration based on the research result.

To allow a visualization of the impact of the efficiency gain, Figure 7 shows the projected service rates ($PS_{j}$, Equation (2)) for the five regions of the country, that is, the percentage of coverage of the population’s service with basic sanitation projected by the estimated efficiency gains compared to the current situation in Brazil (BR). At the same time, the results of the 27 federative units in Brazil are shown in the two scenarios: the projection with efficiency gains, which represents the population served assuming a maximum level of efficiency, when assuming TE=1 on the i-th BSU, specified by $\mu_{i}^{*}$ in Equation (2); different current (real) conditions of service of the sector, which reflects the current level of coverage used to estimate $TE16_{i}$. In this way, the results illustrated in Figure 7 indicate, respectively, how much in percentage of the population is served with water and sewage at the current level of efficiency and how much it would be with the estimated efficiency gains (highlighted projection).

According to projections made about the provision of basic sanitation in the country, there is a potential technical margin for a considerable expansion in the number of people served with this service. In BR, access to piped water and the sewage collection network would raise the level of service to 76.5% of the total population compared to the current 59.9% with water and sewage. Grouping the result by Brazilian region, the southeast region would reach the percentage of 83.8% of the population with water and sewage, the best regional performance in the country. The north region would double the service, although this region still has the worst rate of access to water and sewage (from the current 16.4% of the population, to 53.9% of the population). In this same sense, before with only 38.8% of the population with water and sewage, the northeast region would advance to 64.7% of the population. With the improvement in performance in the northeast, only the north would remain with about half of the population served by water and sewage in Brazil.

The result dynamics shown in Figure 7 provides information about the available resources and production in each Brazilian state and geographic region. The maximization of sectoral productivity shows, for example, that the state of Alagoas would increase from the current 37.5% of basic sanitation coverage level to the level of 62.2%, benefiting 336,184 new people. In the state capital, Maceió/AL, with $tp_{AL}=1021,709$ inhabitants, 218,315 new people would be served by the general sewage system. For the DMU with data from the BSU located in Maceió/AL, with $TE16_{AL}=0.6536$ and $\mu_{AL}=411,972$, Equation (2) results in $PS_{AL}=61.7\%$, that is, a projection of 630,287 people attended before the 411,972 people currently served (40.3%). Similarly, the increase in production of 1628 BSUs is estimated, one by one, projecting them towards the most efficient ones. It is a virtual reference, that is, an optimal situation conditioned to the best use of available resources that also implies more social justice and survival of the BSU.

It is worth noting, in Figure 7, the gaps that predominate in the Brazilian sector due to inefficient BSU and the existing learning potential for better operational guidance to be followed by a BSU, in terms of expanding service. At the intersection by region, the values in Table 5 are found with the best reference standards by geographical coverage. The indispensable search for efficiency is a component of the greatest importance in business competitiveness strategies and, also, in equity of access to basic sanitation services.

Considering the current situation of the 27 federative units in Brazil, 16 states (59.3%) have less than half of the population with access to such services in an integrated manner. From the perspective of increasing efficiency of Brazilian companies, only threewould still remain within worrying reality, namely, RO (36.2%), AC (41.0%), and AP (43.0%), located in the north of the country, as a consequence of the uneven distribution of public services in Brazilian regions where the conditions of poverty persist over time and reproduce the scarcity of the availability of basic infrastructure for the population.

Contributing to the efficient management of basic sanitation in Brazil, the increase in the efficiency level of 1628 companies would provide the following advances. The state of Rondônia (RO) would present the greatest advance in the country, expanding from 67,450 to 184,711 inhabitants served with water and sewage. Currently has $TE16_{RO}$ = 0.2875 given the current inputs and products. The Federal District (DF) would be the first unit of the federation to achieve universal service. In addition, the states of São Paulo (SP), Rio de Janeiro (RJ), and Minas Gerais (MG) would exceed 80% of the population’s service with water and sewage.

The current level of efficiency verified in Brazil (TE16=0.3903) is consistent with other studies applied to the sanitation sector. The study by Ortega et al. [16] calculated an average efficiency for Brazil of 0.3440 for the period 2010–2012, during a survey of 47 countries. Other studies corroborate the potential for an increase in sectoral productivity between 32.6% and 56.9%, showing gaps in the quality of management and best techniques for the maximum possible production in water supply and sanitation by Brazilian companies [21,31]. On the other hand, as they are relative efficiencies, the authors’ research findings should be weighted according to the objectives of each research, characteristics of the DMU and the orientation of the DEA model. However, it appears that the performance in all analyzes points to an average efficiency for Brazil lower and far from 100%.

As mentioned earlier, greater technical efficiency in the sector means a more rational allocation of productive resources. It is necessary to create instruments to encourage the efficiency of BSU, taking into account the results obtained, for example, with the support of effective regulation, whose main practical implication is the need to treat the resource allocation process differently, using based on performance. Only in this way is it possible to make the sector more efficient and sustainable and allow gains with the increase of efficiency to be transferred to users through the expansion of access to water and sewage services by the population, as well as the reduction of tariffs and the provision of a better quality of services provided. Such aspects are essential for the evaluation of business survival alternatives and viable investment projects in a given BSU. In addition, the assessment of competitive positioning is a stimulus to technological innovation that requires investment and professional qualification, as well as public and private institutional support, in particular for smaller BSUs, with restricted market areas in Brazil.

These results are useful in making investment decisions based on BSU capacity, considering the expected return for each situation. For policy purposes, the sector’s efficiency analysis can be used as a strategic indicator to define and evaluate the fulfillment of service delivery goals as part of the global effort of the Sustainable Development Goals (SDGs) that aims to universalize basic sanitation by 2030. Assuming that each BSU signs annual management contracts, establishing quantitative volumes for the provision of services, the study of the projections for the goals under commitment is useful to understand and point out possibilities and references. In addition, policies to stimulate the integrated management of BSU in remote areas and intensify the population’s access to sanitation are important ways to optimize the sector’s results.

The development of an M-DEA model improves regulatory supervision with a view to universal service by the 2030s [26,46]. Thus, the identification of public and private companies operating with inefficiency is essential to guide sector planning to expand coverage of service, in view of the best practices in operation in the BSU observed in the results of this work.

Finally, when considering the factors that influence technical efficiency in the sector, the skills of planning, measurement and execution should be improved in municipalities with less than 100 thousand inhabitants and in stimulating investments in local companies, especially companies that are classified as small company and micro-enterprise that make up 75% of the companies with local/municipal management.

**Figure 8:**
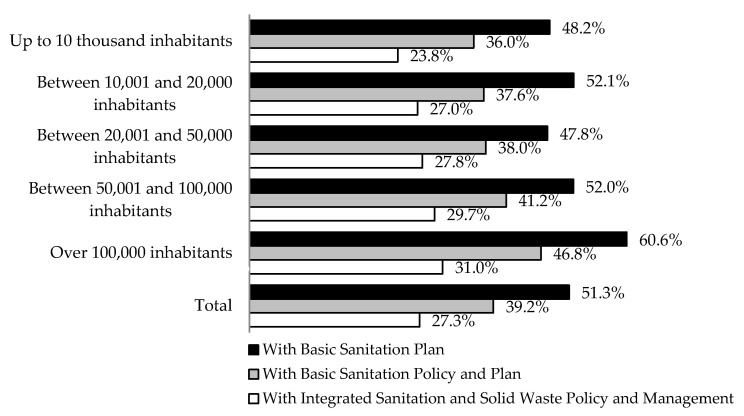
Management context of local and regional basic sanitation companies in Brazil in 2016. Source: Authors’ own elaboration from the NSIS [8].

Although Brazil still suffers a considerable deficit in the access to basic sanitation, the monitoring of the efficiency of the BSU and the regulatory framework instituted in 2007 for the sector provide ways to overcome the deficit and reach the universalization of this service. These paths include strategic BSU planning as an indispensable tool. Figure 8 shows the panorama of sectoral planning considering the location of the BSU and the stratification by size of the municipality by population. There are three important aspects for understanding the challenge to increase efficiency in Brazil, namely: (i) the proportion of units that have Sanitation Plans; (ii) the share of companies that have Sanitation Plans and that operate in municipalities that also have Policies instituted on water and sewage services; and (iii) the ideal situation, in which there is a combination of planning and regulatory instruments on the integration of water, sewage and solid waste management services in the municipalities where the BSUs operate.

A poor planning is identified according to the general level of activity in the preparation of sanitation plans as a strategic tool in the BSU and becomes a risk to the ability to manage infrastructure, financial resources and productivity. The example of BSU in small municipalities with up to 10 thousand inhabitants (Figure 8) points out that less than half of the companies in the sector have a plan to define and evaluate the objectives of expanding the coverage of services to the population in the face of the deficit in the provision of supply services of water and sewage treatment. In general, it is important to highlight that only 51.3% of the BSU in Brazil have sanitation plans and that, as a result of combined management policies and instruments, they correspond to only 39.2% in the country. The vision of integrated management of basic sanitation and solid waste management plans provided for in the sector’s regulatory framework [46] is limited to 27.3% of the municipalities [8]. These are situations that contribute negatively to the low overall performance of the basic sanitation sector in Brazil. This reinforces that regulatory change alone is not enough. This must be accompanied by objective performance assessment instruments to encourage the efficiency of the BSU and the effectiveness of its plans, taking into account the results obtained, the productivity gains and the competitive position in relation to the other companies in the sector, in order to boost the universalization of services in the country.

The results of the present study contribute both to operational management, helping to identify the determinants of low performance and to strategic management, as they provide important information for the decision to expand access to basic sanitation services. The M-DEA approach can be used to develop performance assessments and support resource allocation in Brazil and other countries. This allows the creation of an objective indicator that is more appropriate for measuring the level of efficiency in the integrated management of basic sanitation.

## 4. Conclusions

This article evaluated the efficiency in water supply and sanitation of companies that perform the integrated management of these services in Brazilian municipalities. It is an evaluation using all possible combinations in the subset of inputs and operating results. To achieve this objective, an M-DEA optimization method was implemented to measure the level of technical efficiency by BSU for the years 2008 and 2016, in order to identify changes in the decentralized operation of services (at the municipal level), after the milestone sector’s regulatory framework in 2007. The discussion incorporated, for the first time, the use of M-DEA in the basic sanitation sector to mitigate the lack of standard on which resources and products should be used in the evaluation model of efficient and inefficient companies in Brazil. This methodology can be replicated for the evaluation of BSU in other countries. The results provided empirical evidence on this multiple modeling as an alternative to the conventional DEA structure, as it generates efficiency results that are less sensitive to the choice of inputs and products and, thus, improve the understanding of the operating environment of Brazilian BSUs in the form of a production system.

In summary, the technical efficiency of BSU in most Brazilian municipalities (57.7%) is below the national average in the period studied, signaling the existence of a margin of more than 60% for improvement in the provision of services. It is estimated that the increase in technical efficiency in the BSU would be able to expand the access to piped water and the sewage collection network in Brazil by about 17%, given the current levels of infrastructure, human, and financial resources in the sector. The coverage of the population with basic sanitation in Brazil would go from 59.9% to 76.5% of the population. From a managerial perspective, the empirical application of this study is of great interest to regulators, company managers and public policy makers to contribute to a greater performance of water and sewage services, reducing operating costs and increasing sectoral competitiveness. Under a socio-environmental perspective, the results favor environmental protection and promote the development of a society that strives for human dignity.

For this reason, the identification of benchmarks in the sanitation integrated management sector can strengthen the monitoring of municipal and state sanitation plans and enable the periodic review defined in Brazilian legislation, to recognize their realities and absorb national and international guidelines in this strategy. Furthermore, based on the results, the relevance of building a unified management governance structure and providing transparency regarding the situation of Brazilian municipalities in order to guide investments in the sector, pointing to efficiency in the application of resources, is highlighted, especially in the 20-year horizon defined in the strategic planning (PLANSAB).

The proposed model for measuring efficiency and the results of this research aim to contribute to increase the performance of basic sanitation services in Brazil with a view to assessing problems related to low service coverage and the identification of BSU operating with technical inefficiency. This is fundamental to guide the sector planning in the short and medium terms and for strategic decisions based on performance evidence.

Finally, in view of the evidence findings, it is considered opportune for Brazil to institutionalize the evaluation of technical efficiency as an instrument for monitoring the results of sectoral strategic planning in force until 2033 and meeting the objectives of sustainable development (SDGs) by 2030. The emphasis on studies of efficiency with disaggregated data about integrated management in the basic sanitation sector is particularly important in this regard, as it allows, on the one hand, a better dimensioning of potential waste and, on the other hand, to identify examples of best practices.

For future work, it is recommended to use other approaches to measure technical efficiency, such as two-stage boundary models (with control of contextual variables, e.g., child mortality, GDP, HDI) to estimate managerial efficiency. Furthermore, given the heterogeneity of the DMUs analyzed, it becomes relevant to develop sensitivity analyzes of the estimates to mitigate possible interference from other unobserved variables and in the detection of outliers. In addition, new efficiency measures are encouraged, changing the orientation of the product model to input, to also identify the excessive use of productive resources and potential margins for their reduction.

## Figures and Tables

**Table 1:** Variables used in the literature for sanitation efficiency models applied to DEA used in Brazil.

Author	Resources				Products							Period Studied	BSU	TC(1)	MO(2)	SY(3)	EFF
	OEE	OLF	IEW	IES	DOR	ACW	ACS	VTW	VTS	PSW	PSS						
Macedo [18]	●	-	●	●	-	●	●	-	●	-	-	2004–2015	18	1	I	V	0.8467
Ortega et al. [16]	-	-	-	-	-	-	-	-	-	●	●	2000–2012	47	3	I	V	0.3993
Scaratti et al. [22]	●	-	-	-	-	-	-	●	●	●	●	2010	53	2	P	C	0.7500
Sampaio and Sampaio [24]	●	●	●	●	●	●	●	●	●	●	●	1998–2003	36	1	P	V	0.9460
Motta and Moreira [31]	●	-	-	-	-	●	●	●	●	●	●	1996–2002	104	2	I	C	0.4313
Tupper and Resende [25]	●	●	-	-	-	-	-	-	●	●	●	1996–2000	20	1	P	V	0.7988
Carmo and Távora-Junior [32]	-	●	●	●	-	●	●	●	●	-	-	2000	26	1	P	V	0.9748
													Average (EFF) = 0.7353				
													Standard Deviation (EFF) = 0.2323				

(**●**) Variables used in the literature. (**1**) 1: State/regional, including in the BSU sample of federation units spread across all five Brazilian geographic regions; 2: Municipal/local, contemplating in the BSU sample that operates in municipalities located in all five Brazilian geographic regions; 3: International, covering a comparative study between country performance, including Brazil. (**2**) I: orientation of the model for input; P: model orientation for product. (**3**) C: constant returns to scale of the model; V: variable returns to scale of the model.

**Table 2:** Description of the variables used to evaluate the efficiency index of the sanitation sector in Brazil.

	Variable	Description/Acronym	Unit Measure	Source
Resource	$x_{1}$	Operating exploration expenses (OEE)	R$	NSIS [8,47]
	$x_{2}$	Own labor force (OLF)	People	
	$x_{3}$	Available infrastructure of the extension of the general water (IEW) and sewage (IES) network	Km	
Product	$y_{1}$	Direct operating revenues (DOR)	R$	NSIS [8,47]
	$y_{2}$	Active connections system in the general water (ACW) and sewage (ACS) network	Connection	
	$y_{3}$	Volume of treated water (VTW) and treated sewage (VTS)	1000 m^3^	
	$y_{4}$	Municipal population served with water (PSW) and sewage (PSS) services	People	

**Table 3:** Description of TE scores by administrative network of BSU (basic sanitation unit) using the M-DEA model.

Description	Total			Private Administration			Public Administration		
	TE08	TE16	Δ%	TE08	TE16	Δ%	TE08	TE16	Δ%
**Total**	0.3442	0.3903	13.4	0.3278	0.3802	16.0	0.3476	0.3920	12.8
Lower limit (95% CI)	0.3359	0.3842	14.4	0.3089	0.3649	18.1	0.3384	0.3854	13.9
Upper limit (95% CI)	0.3525	0.3963	12.4	0.3467	0.3956	14.1	0.3568	0.3987	11.7
**Geographic region**									
North	0.3148	0.3652	16.0	0.3207	0.3613	12.7	0.3064	0.3686	20.3
Northeast	0.4031	0.4248	5.38	(*)	0.4683	(*)	0.4031	0.4246	5.33
Southeast	0.3314	0.3931	18.6	0.4535	0.4506	−0.64	0.3289	0.3909	18.9
South	0.3306	0.3635	9.95	0.3186	0.3592	12.7	0.3550	0.3709	4.48
Midwest	0.3218	0.3554	10.4	0.3593	0.4747	32.1	0.3191	0.3387	6.14
**Population range**									
Up to 10 thousand inhabitants	0.2562	0.3340	30.4	0.2582	0.3118	20.8	0.2560	0.3357	31.1
Between 10,001 and 20,000	0.2813	0.3425	21.8	0.2669	0.3107	16.4	0.2870	0.3520	22.6
Between 20,001 and 50,000	0.3242	0.3725	14.9	0.3076	0.3690	20.0	0.3282	0.3732	13.7
Between 50,001 and 100,000	0.3946	0.4288	8.67	0.3753	0.4343	15.7	0.3977	0.4280	7.62
Above 100,000	0.5241	0.5548	5.86	0.5156	0.5535	7.35	0.5256	0.5551	5.61
**Administrative scope**									
Local/municipal management	0.3803	0.4134	8.70	0.4309	0.3754	−12.9	0.4638	0.4068	−12.3
Regional/state management	0.3343	0.3819	14.2	0.3149	0.3390	7.65	0.3588	0.3864	7.69
**Business size ^1^**									
Micro enterprise	0.2931	0.3509	19.7	0.2883	0.2943	2.08	0.3337	0.3544	6.20
Small enterprise	0.3619	0.4095	13.2	0.3486	0.3639	4.39	0.4245	0.4076	−3.98
Medium-sized enterprise	0.4668	0.4996	7.03	0.4654	0.4670	0.34	0.4982	0.4999	0.34
Large enterprise	0.6698	0.6702	0.06	0.8627	0.6544	−24.1	0.7267	0.6593	−9.27
**Municipal infant mortality rate under 5 years ^2^**									
Municipality low mortality	0.3509	0.4006	14.16	0.3418	0.3863	13.0	0.3527	0.4032	14.3
Municipality average mortality	0.3328	0.3542	6.43	0.2999	0.3571	19.1	0.3401	0.3538	4.03
Municipality high mortality	0.2742	0.3302	20.42	0.1663	0.2411	45.0	0.2796	0.3387	21.1

Note: (*) No BSU for this classification. ^1^ Defined according to the number of own employees (OE) in fourcategories of enterprises adopted by SEBRAE: 1-micro-company (OE ≤ 19); 2-small (20 ≤ OE ≤ 99); 3-average (100 ≤ OE ≤ 499); and, 4-large (OE ≥ 500). ^2^ According to WHO, the infant mortality rate for children under 5 years old (IMR5) is divided into three categories: 1-low (IMR5 < 20); 2-average (20 ≤ IMR5 < 50); and, 3-high (IMR5 ≥ 50).

**Table 4:** Summarizes the central trend of the difference in values in percentage points (p.p.) by dimension in 2008 and 2016.

2008				2016		
Category	Average	Median	MW Test (1)	Average	Median	MW Test (1)
D1	1.98	1.82	63,910 *	1.18	1.34	157,899
D2	7.31	6.45	51,944 ***	4.31	4.12	165,411 ***
D3	21.7	19.8	12,688 ***	19.3	18.0	36,010 ***
D4	4.60	6.03	60,986 ***	3.15	3.71	214,476 ***
D5	33.5	33.5	1280 ***	28.5	25.0	3086 ***
D6	2.29	1.78	93,869 *	4.91	3.59	171,925 ***

(1) MW: Mann–Whitney U statistic value. Note: *** and * denote significance at p < 0.001; and p < 0.05, respectively.

**Table 5:** BSU more and less efficient by Brazilian region in 2016.

Geographical Location	TE16 Maximum	BSU Name and Municipality/FU	TE16 Minimum	BSU Name and Municipality/FU
BR	0.9949	EMBASA—Salvador/BA	0.0723	Prefeitura Munic. Campos N. Paulista/SP
N	0.7907	Manaus Ambiental—Manaus/AM	0.1864	SANEATINS—Augustinópolis/TO
NE	0.9949	EMBASA—Salvador/BA	0.1341	CAERN—José da Penha/RN
SE	0.9750	COPASA—Belo Horizonte/MG	0.0723	Prefeitura Munic. de Campos Novos/SP
S	0.8596	Prefeitura Municipal—Tupandi/RS	0.1340	CASAN—Rancho Queimado/SC
MW	0.9920	CAESB—Brasília/DF	0.1767	SANEAGO—Mimoso de Goiás/GO

Legend: BR = Brazil; N = North Region; NE = Northeast Region; SE = Southeast Region; S = South Region; MW = Midwest Region; FU = Federative Unit.

## Appendix A

**Table A1:** Efficiency scores of the 20 basic sanitation companies with the best and worst results (ordered in descending order).

More Efficient.						Less Efficient					
Ord.	BSU	FU	COV.	ADM.	*TE*	Ord.	BSU	FU	COV.	ADM.	*TE*
2016											
1	EMBASA	BA	R	APU	0.9949	1609	SANEATINS	TO	R	APR	0.1864
2	CAESB	DF	R	APU	0.9920	1610	SANESUL	MS	R	APU	0.1853
3	COPASA	MG	R	APU	0.9750	1611	CORSAN	RS	R	APU	0.1850
4	CEDAE	RJ	R	APU	0.9442	1612	SANEAGO	GO	R	APU	0.1841
5	CEDAE	RJ	R	APU	0.9233	1613	SANESUL	MS	R	APU	0.1828
6	COMPESA	PE	R	APU	0.8712	1614	SANEAGO	GO	R	APU	0.1767
7	CEDAE	RJ	R	APU	0.8679	1615	SABESP	SP	R	APU	0.1759
8	SAAE	SP	L	APU	0.8604	1616	COPASA	MG	R	APU	0.1758
9	PMT	RS	L	APU	0.8596	1617	PMER	SC	L	APU	0.1729
10	CEDAE	RJ	R	APU	0.8563	1618	SABESP	SP	R	APU	0.1704
11	SABESP	SP	R	APU	0.8364	1619	PMI	SC	L	APU	0.1699
12	SABESP	SP	R	APU	0.8336	1620	SABESP	SP	R	APU	0.1652
13	COMPESA	PE	R	APU	0.8323	1621	COPASA	MG	R	APU	0.1649
14	SANEPAR	PR	R	APR	0.8289	1622	PMJ	SP	L	APU	0.1633
15	AG	MS	L	APR	0.8192	1623	SABESP	SP	R	APU	0.1627
16	CAGEPA	PB	R	APU	0.8096	1624	SAMASA	SC	L	APU	0.1513
17	MA	AM	L	APR	0.7907	1625	COPASA	MG	R	APU	0.1432
18	SANEAGO	GO	R	APU	0.7857	1626	CAERN	RN	R	APU	0.1341
19	SANEPAR	PR	R	APR	0.7808	1627	CASAN	SC	R	APU	0.1340
20	SANEAR	MT	L	APU	0.7801	1628	PMCNP	SP	L	APU	0.0723
**2008**											
1	COPASA	MG	R	APU	0.9903	982	SANEPAR	PR	R	APR	0.1667
2	CAESB	DF	R	APU	0.9737	983	SANEPAR	PR	R	APR	0.1663
3	EMBASA	BA	R	APU	0.9555	984	SABESP	SP	R	APU	0.1658
4	ADA	AM	L	APR	0.8829	985	SAAE	BA	L	APU	0.1657
5	CAGECE	CE	R	APU	0.8795	986	SABESP	SP	R	APU	0.1639
6	CEDAE	RJ	R	APU	0.8495	987	SANEAGO	GO	R	APU	0.1592
7	SANEPAR	PR	R	APR	0.8425	988	SANEPAR	PR	R	APR	0.1588
8	COPASA	MG	R	APU	0.8270	989	SANEAGO	GO	R	APU	0.1584
9	COMPESA	PE	R	APU	0.8187	990	SANEAGO	GO	R	APU	0.1573
10	SEMAE	SP	L	APU	0.8025	991	SANEPAR	PR	R	APR	0.1549
11	SABESP	SP	R	APU	0.7796	992	SAAE	PR	L	APU	0.1529
12	CAEMA	MA	R	APU	0.7670	993	SABESP	SP	R	APU	0.1462
13	COPASA	MG	R	APU	0.7616	994	SABESP	SP	R	APU	0.1459
14	CAGEPA	PB	R	APU	0.7587	995	PM	RS	L	APU	0.1435
15	CEDAE	RJ	R	APU	0.7567	996	SAAE	RO	L	APU	0.1389
16	EMASA	SC	L	APU	0.7565	997	SABESP	SP	R	APU	0.1384
17	DMAE	RS	L	APU	0.7549	998	SABESP	SP	R	APU	0.1304
18	COPASA	MG	R	APU	0.7520	999	SABESP	SP	R	APU	0.1277
19	CESAN	ES	R	APU	0.7408	1000	SABESP	SP	R	APU	0.1274
20	SANEPAR	PR	R	APR	0.7384	1001	SANEAGO	GO	R	APU	0.1070

Legend: The column (“BSU”) identifies the basic sanitation company’s acronym. The column (“FU”) identifies the federative units of Brazil. In the Coverage column (“COV”), the annotation “R” corresponds to the company with Regional/State Administration; and “L” refers to the company with Local/Municipal Administration. The column (“ADM”) identifies the company’s administrative network, being “APU” of public scope and “APR” of private scope.

**Table A2:** Descriptive statistics of input and output variables, 2008 and 2016.

Variables		Minimum	Maximum	Mean	SD
2008 (*n* = 1008)					
**Inputs**	$x_{1}$	165,865.12	1529,717,412.04	17,416,790.37	71,318,066.52
	$x_{2}$	1.00	4088.00	76.11	240.67
	$x_{3}$	2.05	14,066.00	404.58	1044.52
**Outputs**	$y_{1}$	80,812.78	3930,372,386.63	25,551,357.32	146,735,795.45
	$y_{2}$	563.00	1464,479.00	32,873.41	92,239.65
	$y_{3}$	58.39	1407,857.00	10,453.75	52,715.68
	$y_{4}$	1559.00	11,199,584.00	134,813.71	508,222.86
**2016** (***n* = 1641**)					
**Inputs**	$x_{1}$	60,647.63	2863,527,437.74	17,994,493.91	94,438,463.88
	$x_{2}$	1.00	4897.00	66.28	231.10
	$x_{3}$	4.80	38,363.64	417.40	1369.48
**Outputs**	$y_{1}$	5345.86	5524,767,969.68	26,861,903.02	178,360,824.24
	$y_{2}$	220.00	5683,870.00	36,360.26	167,613.01
	$y_{3}$	18.00	1416,456.00	8915.05	44,494.30
	$y_{4}$	660.00	23,546,671.00	129,259.83	718,801.52

SD = Standard Deviation.

**Table A3:** Correlation matrix between input and output variables, 2008 and 2016.

2008 (*n* = 1008)							
Variables	$x_{1}$	$x_{2}$	$x_{3}$	$y_{1}$	$y_{2}$	$y_{3}$	$y_{4}$
$x_{1}$	1	0.874 **	0.894 **	0.953 **	0.922 **	0.929 **	0.970 **
$x_{2}$	0.874 **	1	0.878 **	0.803 **	0.876 **	0.814 **	0.878 **
$x_{3}$	0.894 **	0.878 **	1	0.805 **	0.966 **	0.793 **	0.914 **
$y_{1}$	0.953 **	0.803 **	0.805 **	1	0.856 **	0.977 **	0.960 **
$y_{2}$	0.922 **	0.876 **	0.966 **	0.856 **	1	0.851 **	0.956 **
*y*_3_	0.929 **	0.814 **	0.793 **	0.977 **	0.851 **	1	0.955 **
$y_{4}$	0.970 **	0.878 **	0.914 **	0.960 **	0.956 **	0.955 **	1
**2016** (***n* = 1641**)							
**Variables**	$x_{1}$	$x_{2}$	$x_{3}$	$y_{1}$	$y_{2}$	$y_{3}$	$y_{4}$
	1	0.903 **	0.955 **	0.968 **	0.959 **	0.715 **	0.970 **
$x_{2}$	0.903 **	1	0.930 **	0.862 **	0.862 **	0.759 **	0.866 **
$x_{3}$	0.955 **	0.930 **	1	0.934 **	0.957 **	0.718 **	0.947 **
$y_{1}$	0.968 **	0.862 **	0.934 **	1	0.956 **	0.777 **	0.988 **
$y_{2}$	0.959 **	0.862 **	0.957 **	0.956 **	1	0.634 **	0.985 **
$y_{3}$	0.715 **	0.759 **	0.718 **	0.777 **	0.634 **	1	0.721 **
$y_{4}$	0.970 **	0.866 **	0.947 **	0.988 **	0.985 **	0.721 **	1

Note: ** denote significance at p < 0.01.

**Table A4:** Comparison of the company’s inputs and products with the highest TE value (benchmark) and the lowest TE value.

Var.	Resources/Products	Highest TE16	Lowest TE16
TE	Technical Efficiency (0 to 1)	0.9949	0.0723
OEE	Operating exploration expenses (R$ 1000)	653,297.33	5000.07
OLF	Own labor force (people)	1447.00	252.00
IEW	Available infrastructure of the extension of the general water network (km)	5358.67	42.00
IES	Available infrastructure of the extension of the general sewage network (km)	3506.06	65.00
DOR	Direct operating revenues (R$ 1000)	1064,040.20	171.73
ACW	Active connections system in the general water network (Connection)	499,119.00	1451.00
ACS	Active connections system in the general sewage network (Connection)	496,948.00	1451.00
VTW	Volume of treated water (1000 m^3^)	280,019.96	246.10
VTS	Volume of treated sewage	144,408.03	40.00
PSW	Municipal population served with water services (people)	2660,120.00	4808.00
PSS	Municipal population served with sewage services (people)	2313,807.00	4808.00

**Table A5:** Observations removed from the sample by using the super-efficiency (SM) model to identify outliers, according to Banker and Chang [52].

Ord.	Municipality Code	Municipality Name	SM	Ord.	Municipality Code	Municipality Name	SM
2008				2016			
1	320520	Vila Velha	4.15	1	421570	Santo Amaro da Imperatriz	4.83
2	230370	Caucaia	3.91	2	313652	José Gonçalves de Minas	4.34
3	330455	Rio de Janeiro	3.79	3	355030	São Paulo	3.51
4	320510	Viana	3.1	4	330455	Rio de Janeiro	3.34
5	230970	Pacatuba	2.27	5	270760	Quebrangulo	2.96
6	412862	Alto Paraíso	2.07	6	431445	Pinhal	2.72
7	353160	Monte Castelo	2.02	7	241142	Santana do Seridó	2.69
				8	411915	Pinhais	2.55
				9	352560	João Ramalho	2.49
				10	410400	Campina Grande do Sul	2.39
				11	432215	Tunas	2.07
				12	230440	Fortaleza	2.03
				13	210530	Imperatriz	2.01
